# Family cohesion and adaptability in heart failure: an APIMeM analysis of symptom perception and spousal caregiving on patient self-care

**DOI:** 10.3389/fmed.2026.1684435

**Published:** 2026-06-19

**Authors:** Yan Yang, Jianhua Shi, Xiaoying Gu

**Affiliations:** Department of Cardiology, Rugao Hospital of Traditional Chinese Medicine, Nantong, China

**Keywords:** actor-partner interdependence model, caregiving contribution, chronic heart failure, family cohesion and adaptability, self-care ability, symptom perception

## Abstract

**Objective:**

To examine the actor-partner interdependence mediation effect of efamily cohesion and adaptability on self-care ability in patients with chronic heart failure (CHF) and their spouses, mediated through symptom perception and caregiving contribution.

**Methods:**

A cross-sectional survey was conducted among 212 CHF inpatients and their spouses from Rugao Hospital of Traditional Chinese Medicine, Nantong. Core variables were assessed using: Family Adaptability and Cohesion Scale (FACES-II), Heart Failure Somatic Perception Scale (HFSPS), European Heart Failure Self-Care Behavior Scale for Caregivers (EHFScBS-C), Self-Care of Heart Failure Index (SCHFI). Actor-Partner Interdependence Mediation Model (APIMeM) was employed to analyze actor effects and partner effects.

**Results:**

The family function scores of CHF patients and their spouses were (102.36 ± 18.24) and (98.75 ± 16.83) respectively. The total score of HFSPS for patients was (52.71 ± 12.38), and the total score of EHFScBS-C for spouses was (64.18 ± 14.79). The total score of SCHFI for patients was (155.73 ± 43.30), among which the management dimension was the weakest with a score of (48.67 ± 13.76). In the subject effect analysis, the direct effect of family function on self-care ability accounted for 38.5%, the indirect path through symptom perception accounted for 35.3%, and the indirect path through care contribution accounted for 26.2%. In the object effect analysis, the direct effect of the spouse’s family function on the patient’s self-care ability accounted for 40.0%, the indirect path through the patient’s symptom perception accounted for 32.3%, and the indirect path through the spouse’s care contribution accounted for 27.7%.

**Conclusion:**

Family cohesion and adaptability are associated with lower levels of symptom burden and greater spousal care contributions, and are both linked to better self-care ability in patients with CHF. Clinical interventions should consider the patient-spouse dyad as a whole. By improving family adaptability and providing targeted support for spouses in managing daily behaviors, patient self-care ability can be enhanced.

## 1 Introduction

Chronic Heart Failure (CHF) is a severe and increasingly prevalent chronic condition. Studies estimate that the global prevalence of heart failure is approximately 64 million (1). Its long-term management and rehabilitation outcomes heavily depend on the collaborative efforts of patients and their primary caregivers (typically spouses) (2). In disease management, patients’ self-care ability—encompassing symptom recognition, medication adherence, lifestyle adjustments, and timely medical consultation—is a core determinant of improved prognosis, reduced rehospitalization risk, and enhanced quality of life (3, 4).

Family cohesion and adaptability, as core indicators of family system functioning (5), profoundly reflect emotional bonds among members (cohesion) and their capacity to adjust under stress (adaptability) (6). Strong family cohesion and adaptability provide robust emotional support and a collaborative environment, which have been demonstrated to facilitate health behavior establishment and management efficacy in chronic disease patients (7). For CHF patients, spousal involvement and support are critical for sustaining effective self-care (8). Conversely, patients’ illness perception, particularly their assessment of symptom burden (e.g., dyspnea, fatigue, edema), directly influences disease cognition, emotional responses, and subsequent management behaviors (9). Elevated symptom perception may trigger fear and avoidance behaviors, thereby undermining motivation and efficacy in self-care (10).

Existing studies indicate that ‘illness perception correlates with self-care ability, while family cohesion and adaptability significantly impact disease management and well-being. However, prior research predominantly focuses on single-subject effects (e.g., patient-level pathways) or simple correlations between variables. Key gaps include: Lack of dyadic perspective: It remains unclear how patient-perceived and spouse-perceived family functioning independently influence patients’ illness perception and self-care ability (actor effects) or how one partner’s perceived family functioning affects the other’s outcomes (partner effects). Unclear mediation mechanisms: The specific pathways through which patients’ illness perception and spouses’ caregiving contributions connect family cohesion/adaptability to self-care ability—whether independent or interactive—are poorly understood. Absence of integrated models: A framework integrating patient-reported family function, illness perception, and self-care ability with spouse-reported family function and caregiving contributions is needed to capture dynamic dyadic interactions.

**Figure 1:**
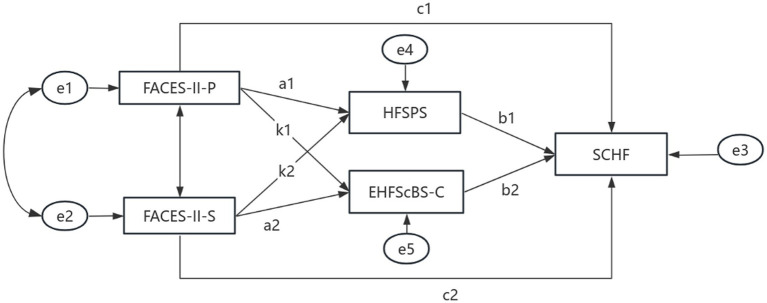
Hypothesized model of relationships between family cohesion/adaptability, symptom perception, spousal caregiving contribution, and patient self-care ability (a, b, c = actor effects; k = partner effects; e1-e5 = error terms). FACES-II-P, Family Adaptability and Cohesion Evaluation Scales II—Patient Version; FACES-II-S, Family Adaptability and Cohesion Evaluation Scales II—Spouse Version; HFSPS, Heart Failure Somatic Perception Scale; EHFScBS-C, European Heart Failure Self-care Behavior Scale—Caregiver Version; SCHF, Self-Care of Heart Failure Index.

To dissect the complex chain linking dyad-perceived family cohesion/adaptability to patients’ self-care ability via patients’ intrinsic perception (illness perception) and spouses’ extrinsic behaviors (caregiving contributions), this study innovatively applies the Actor-Partner Interdependence Mediation Model (APIMeM) (11). As illustrated in Figure 1.

Independent variables: Patient-rated family cohesion/adaptability (FACES-II-P), Spouse-rated family cohesion/adaptability (FACES-II-S). Mediators: Patient symptom perception (HFSPS), Spouse caregiving contribution (EHFScBS-C). Dependent variable: Patient self-care ability (SCHFI). The model mainly verifies the following three hypotheses.

*H1*: Actor effects: FACES-II-P → HFSPS → SCHFI; FACES-II-S → EHFScBS-C → SCHFI.

*H2*: Partner effects: FACES-II-P → EHFScBS-C → SCHFI; FACES-II-S → HFSPS → SCHFI.

*H3*: Dual mediation: HFSPS and EHFScBS-C jointly mediate the family function → self-care pathway, with significant differences in actor/partner effect contributions and direct/indirect effect strengths.

This study aims to reveal multi-path mechanisms through which family functioning influences self-care ability from a patient-spouse dyadic perspective. Findings will inform the development of personalized, family-integrated interventions targeting dyadic interaction patterns, symptom perception alleviation, and optimized spousal contributions to enhance CHF self-care.

## 2 Research subjects and methods

### 2.1 Research subjects

This study employed convenience sampling to recruit hospitalized patients with CHF and their spouses from the Department of Cardiology, Rugao Hospital of Traditional Chinese Medicine, Nantong, between January 2024 and April 2025. A total of 212 valid dyads were included. Patients Inclusion Criteria ① Met diagnostic criteria of Chinese Guidelines for the Diagnosis and Management of Heart Failure, NYHA class II–III; ② Age ≥18 years; ③ Married with spouse as primary caregiver. Patients Exclusion Criteria: ① Comorbid with malignancies, end-stage diseases (e.g., severe hepatic/renal failure), or psychiatric disorders; ② Cognitive impairment (MMSE <24) or hearing loss affecting participation (12). Spouses Inclusion Criteria: ① Independent in daily living and caregiving abilities; ② Age ≥18 years; ③ Designated as primary caregiver by the patient; Exclusion Criteria: ① History of severe cardiovascular/cerebrovascular diseases, psychiatric disorders, or other chronic conditions requiring long-term treatment; ② Cognitive/communication barriers impairing questionnaire completion. The sample size is determined based on the sample size requirements of the structural equation model (SEM) for APIMeM. Ledermann et al. (13) sample size should be 10 to 20 times the number of dimensions of the measured variables. The core scale of this study includes the following dimensions: FACES-II (2 dimensions), HFSPS (1 dimension), EHFScBS-C (2 dimensions), and SCHFI (3 dimensions), totaling 10 measurement dimensions. A minimum of 100 to 200 pairs of samples is required. Considering a 20% risk of invalid or dropped questionnaires, the final sample size was determined to be 212 pairs, which meets the methodological requirements. This study was approved by the Ethics Committee of Rugao Hospital of Traditional Chinese Medicine, Nantong City (Approval Number: RGSZYYLL2024058), and all patients have signed the informed consent form.

### 2.2 Research method

#### 2.2.1 Research instruments

##### 2.2.1.1 General Information Questionnaire

This study utilized self-designed questionnaires, which were divided into patient and spouse versions. The specific contents included sociodemographic data: age, gender, BMI, educational level, occupational status, personal annual income, and daily care time provided by the spouse (in hours); disease-related data: NYHA heart function classification (Class II/III), LVEF (%), duration since heart failure diagnosis (in years), number of hospitalizations in the past year, presence of diabetes, and current use of diuretics/*β*-blockers/ARNI drugs.

##### 2.2.1.2 Family Adaptability and Cohesion Evaluation Scales

**Figure 2:**
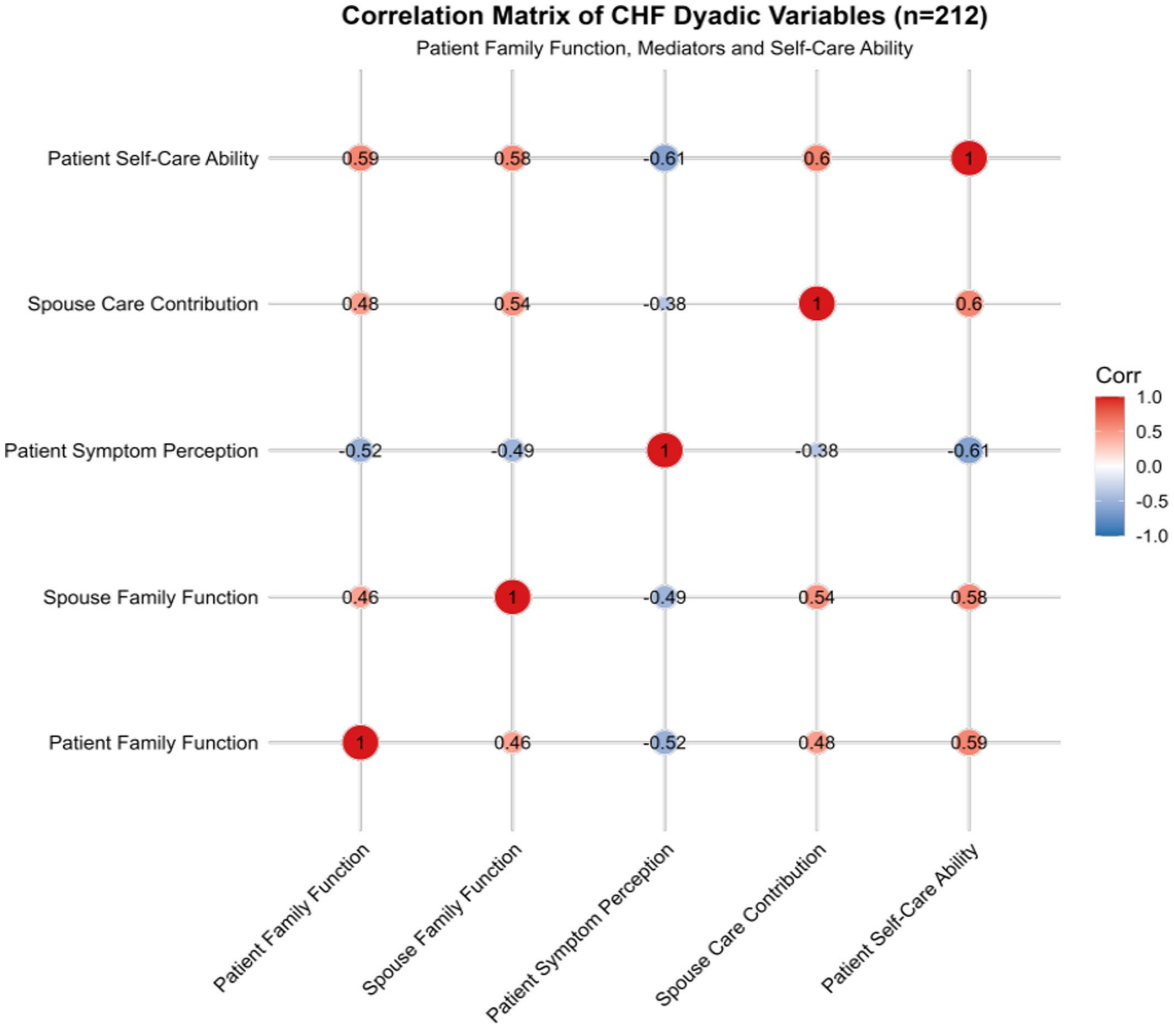
Correlation matrix of CHF dyadic family function, mediators and self-care ability. Circle size represents the strength of correlation; color indicates direction (blue = negative, red = positive). All correlations were statistically significant (*p* < 0.01).

The Chinese version translated by Fei et al. used. This 30-item scale comprises two dimensions. This scale consists of two dimensions: the Intimacy Dimension (16 items) assesses the degree of emotional connection among family members; the Adaptability Dimension (14 items) evaluates the family’s ability to adapt to external pressures. The scale contains a total of 30 items and uses a 5-point Likert scale (1 = “Never” to 5 = “Always”). The scores for each dimension are the sum of the item scores, and the total score of the scale ranges from 30 to 150 points. The higher the total score, the better the level of family intimacy and adaptability. The Cronbach’s *α* coefficient for the Chinese version of this scale in the study population for reliability verification: Intimacy Dimension = 0.89, Adaptability Dimension = 0.87 (Figure 2).

##### 2.2.1.3 Heart Failure Somatic Perception Scale

The HFSPS patients’ perceived symptom burden. This scale consists of 18 items, covering the core symptom clusters of heart failure (such as dyspnea, fatigue, edema, etc.). It uses a 6-point Likert scale (0–5 points), where 0 indicates no symptom and 5 indicates extreme distress. The total score ranges from 0 to 90, with higher scores indicating a stronger perception of symptom distress. In this study, the Cronbach’s *α* coefficient of the scale was 0.862, suggesting good internal consistency.

##### 2.2.1.4 European Heart Failure Self-Care Behavior Scale—Caregiver version (Chinese)

The Chinese version of the Heart Failure Caregiver Contribution Scale (EHFScBS-C), which was revised by Italian scholars Durante et al. in 2020 based on the European Heart Failure Self-Care Behavior Scale, was adopted (14, 15). The scale consists of two dimensions, including medical-related contributions (5 items) and life behavior contributions (4 items). It uses a 5-point Likert scale (1 = “never” to 5 = “always”), with an original total score ranging from 9 to 45. The standardized total score = (original score-9)/36 × 100, and the standardized score range is 0–100. The higher the score, the stronger the caregiver’s contribution to the patient’s self-care.

##### 2.2.1.5 Self-Care of Heart Failure Index

The Chinese version of the Heart Failure Self-Care Behavior Scale, which was developed by Riegel et al. localized by Chen Wei’s team (16), was adopted. The scale consists of three dimensions and 22 items, including self-care maintenance (10 items), self-care management (6 items), and self-care confidence (6 items). The independent standardized score of each dimension is calculated as (actual raw score—theoretical minimum score)/theoretical score range × 100. The total score ranges from 0 to 300, which is the sum of the standardized scores of the three dimensions. The higher the score, the better the self-care ability. Among them, a total score < 145 indicates a lower level of self-care behavior, 145–192 indicates a medium level of self-care behavior, and > 192 indicates a higher level of self-care behavior.

#### 2.2.2 Data collection

This study was conducted in the Department of Cardiology at Rugao Hospital of Traditional Chinese Medicine in Nantong City. The questionnaires were administered by the head nurses of the cardiology department, who had undergone unified training and possessed at least 5 years of experience in heart failure care, to patients 24–48 h before discharge, during the stable period of the disease. A standardized informed consent process was followed: the research purpose and the requirements for completing the questionnaire were explained to the patients and their spouses separately; it was emphasized that both parties should answer independently in physically separated spaces (with a distance of at least 2 m or separated by a screen). The questionnaire combination included: for the patients, a general information form, FACES-II, HFSPS, SCHFI; for the spouses, a general information form, FACES-II, EHFScBS-C. Quality control measures comprised on-site supervision of the filling process (average time: 28 ± 5 min for patients, 18 ± 3 min for spouses), and immediate verification of the completeness of the items. For those with more than 10% of items missing, they were required to complete the missing parts. A total of 230 pairs of questionnaires were distributed in this study, and 212 pairs were effectively retrieved, with an effective rate of 92.17%; the main reasons for invalid questionnaires were fluctuations in the patients’ conditions (8 pairs).

#### 2.2.3 Statistical methods

Data analysis was conducted using SPSS 26.0 and AMOS 26.0 software. Measurement data conforming to a normal distribution were expressed as $\bar{x}\pm s$ , and count data were described by frequency (proportion). Initially, common method bias was evaluated using Harman’s single-factor test (the first factor explained rate < 30%). Pearson correlation analysis was employed to examine the correlation between core variables. The mediator effect of the actor-partner interdependence model was tested by constructing the APIMeM model. Maximum likelihood estimation was utilized for parameter estimation. The model evaluation criteria were CFI/TLI > 0.90, RMSEA < 0.08, SRMR < 0.08. Bootstrap sampling was conducted 5,000 times for bias correction. The significance of the effect was determined by whether the 95% confidence interval excluded 0. The effect size was decomposed by calculating the contribution rate of six indirect paths (including actor, partner, and cross-mediators). The statistical significance level was set at *α* = 0.05 (two-tailed test). Control variables included age, NYHA classification, and disease duration.

## 3 Results

### 3.1 General characteristics of patients and spouses

A total of 212 patient-spouse dyads were included. The mean age was 66.2 ± 5.3 years for patients and 62.4 ± 7.1 years for spouses. Detailed characteristics are summarized in Table 1.

**Table 1:** General information of patients and their spouses (*n* = 212).

Item	Patients (%)	Spouses (%)	Item	Patients (%)	Spouses (%)
Age (years)			BMI(kg/m^2^)		
<60	50(23.6)	62(29.2)	<18.5	32(15.1)	29(13.7)
≥60	162(76.4)	150(70.8)	18.5 ~ 23.9	126(59.4)	148(69.8)
Gender			24 ~ 29.9	44(20.8)	32(15.1)
Male	134(63.2)	78(36.8)	≥30	10(4.7)	3(1.4)
Female	78(36.8)	134(63.2)	NYHA Class		
Education level			Class II	138(65.1)	–
Primary or below	46(21.7)	40(18.9)	Class III	74(34.9)	–
Junior/senior high school	108(50.9)	118(55.7)	Duration of HF diagnosis (years)		
College or above	58(27.4)	54(25.5)	≤1	52(24.5)	–
Employment Status			>1	160(75.5)	–
Employed	28(13.21)	35(16.51)	Hospitalizations (past year)		
Unemployed/retired	184(86.79)	177(83.49)	≤1	86(40.6)	–
Annual income (10,000 yuan)			≥2	126(59.4)	–
<3	68(32.1)	62(29.2)	Diabetes comorbidity		
3 ~ 6	98(46.2)	100(47.2)	Yes	80(37.7)	–
>6	46(21.7)	50(23.6)	No	132(62.3)	–
Daily caregiving hours			Current medications		
<4	–	56(26.4)	Diuretics	192(90.6)	–
4 ~ 6	–	108(50.9)	β-blockers	180(84.9)	–
>6	–	48(22.6)	ARNI	66(31.1)	–
LVEF grade					
<40%	86(40.6)	–			
40% ~ 49%	102(48.1)	–			
≥50%	24(11.3)	–			

### 3.2 Common method bias test

The Kaiser-Meyer-Olkin (KMO) measure of sampling adequacy was 0.82 for patient data and 0.79 for spouse data. Bartlett’s test of sphericity yielded χ^2^ = 2103.7 (patients) and χ^2^ = 1895.4 (spouses), with both *p* < 0.001, confirming suitability for principal component analysis (PCA). To assess potential common method bias, Harman’s single-factor test was performed separately for patient and spouse data through unrotated PCA. For patient scales (FACES-II-P, HFSPS, SCHFI), 18 factors with eigenvalues > 1 were extracted, with the first factor explaining 19.27% of the variance. For spouse scales (FACES-II-S, EHFScBS-C), 16 factors with eigenvalues >1were extracted, with the first factor explaining 15.84% of the variance. All initial factors explained < 40% of the variance, indicating no significant common method bias in this study.

### 3.3 Scores of FACES-II, HFSPS, EHFScBS-C, and SCHFI of CHF patients and their spouses CHF

CHF patients reported a total score of 102.36 ± 18.24on the FACES-II, while their spouses reported 98.75 ± 16.83. HFSPS total score was 52.7 ± 12.38. Spouses scored higher in medical behavior contribution (68.35 ± 10.62) than in lifestyle management contribution (59.73 ± 12.47), with a standardized total contribution of 64.1 ± 14.79. Patients’ self-care ability, measured by SCHFI, had a total score of 155.73 ± 43.30, with the management dimension (48.67 ± 13.76) identified as the weakest area shown in Table 2.

**Table 2:** Scale scores of CHF patients and spouses (*n* = 212).

Scale and dimensions	Patient scores	Spouse scores
FACES-II (family function)		
Cohesion	64.32 ± 9.84	62.73 ± 10.23
Adaptability	38.10 ± 8.42	36.11 ± 7.95
Total score (range: 30–150)	102.36 ± 18.24	98.75 ± 16.83
HFSPS (symptom perception)		
Symptom distress (range: 0–90)	52.71 ± 12.38	
EHFScBS-C (caregiving contribution)		
Medical behavior contribution	–	68.35 ± 10.62
Lifestyle management contribution	–	59.73 ± 12.47
Standardized total (0–100)	–	64.18 ± 14.79
SCHFI (self-care ability)		
Maintenance (range: 0–100)	55.82 ± 14.33	–
Management (range: 0–100)	48.67 ± 13.76	–
Confidence (range: 0–100)	51.24 ± 15.21	–
Total standardized (range: 0–300)	155.73 ± 43.30	–

### 3.4 Correlations among CHF patients’ and spouses’ family adaptability and cohesion, illness perception, spousal caregiving contribution, and patients’ self-care ability

Positive correlations were observed between FAC and self-care ability in both CHF patients and spouses (*p* < 0.001). Conversely, FAC and patient symptom perception were negatively correlated (*p* < 0.001), while FAC and spousal caregiving contribution were positively correlated (*p* < 0.001). Patients’ symptom perception and self-care ability also showed a significant negative correlation (*p* < 0.001). Details are provided in Table 3 and Figure 1.

**Table 3:** Correlations among family function, mediating variables, and self-care ability (*n* = 212, r).

Item	①	②	③	④	⑤
① Patient’s FAC	1	–	–	–	–
② Spouse’s FAC	0.462^a^	1	–	–	–
③ Patient’s symptom perception	−0.521^a^	−0.487^a^	1	–	–
④ Spouse’s caregiving contribution	0.483^a^	0.538^a^	−0.380^a^	1	–
⑤ Patient’s self-care ability	0.593^a^	0.576^a^	−0.612^a^	0.597^a^	1

^a^*P* < 0.01.

### 3.5 Constraint testing and fit indices of APIMeM model

**Figure 3:**
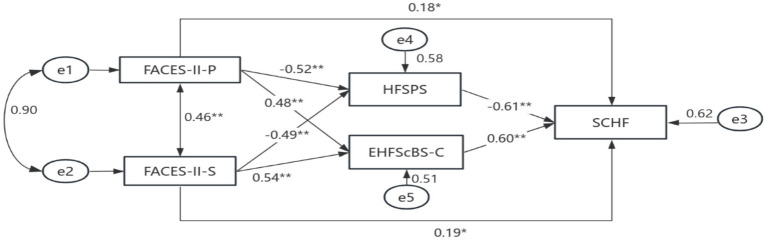
Path analysis model of family cohesion/adaptability, symptom perception, spouse care contribution and self-care agency. FACES-II-P, Family Adaptability and Cohesion Evaluation Scales II—Patient Version; FACES-II-S, Family Adaptability and Cohesion Evaluation Scales II—Spouse Version; HFSPS, Heart Failure Somatic Perception Scale; EHFScBS-C, European Heart Failure Self-care Behavior Scale—Caregiver Version; SCHF, Self-Care of Heart Failure Index. *p < 0.05; **p < 0.01.

An APIMeM was constructed to examine the mediating pathways through which family function (FACES-II) influences self-care ability via symptom perception (HFSPS) and caregiving contribution (EHFScBS-C) (Figure 3). The invariance test of indistinguishability showed that the family function (FACES-II) reported by patients and their spouses had measurement invariance (ΔCFI = 0.008), and there was no gender difference in the subject effect and object effect (*p* = 0.214), indicating that the data conformed to the characteristics of indistinguishable dyads (*p* > 0.20), and the path coefficient constraint model should be adopted. Based on indistinguishability requirements, three sets of path coefficients were constrained to equality:

(1) Actor Effect: a1 = a2.

Path from patient’s family function (X1) → patient’s symptom perception (M1) equals

Path from spouse’s family function (X2) → spouse’s caregiving contribution (M2).

(2) Partner Effect: k1 = k2
  Path from X1 → M2 equals Path from X2 → M1.

Mediation effect: b1 = b2

Path from M1 → self-care ability (Y) equals Path from M2 → Y. All fit indices reached the recommended thresholds, indicating excellent model fit (Table 4).

**Table 4:** Fit indices of the APIMeM model.

Fit index	Criterion	Current model
χ^2^/df	<3	1.42
RMSEA (90%CI)	<0.08	0.029 (0.015–0.042)
CFI	>0.90	0.992
TLI	>0.90	0.953
SRMR	<0.08	0.036
GFI	>0.90	0.971
AGFI	>0.90	0.916

### 3.6 Actor-partner interdependence mediation effects

The *k*-value test (*k* = 0.64, 95% CI: 0.543–1.211, including 1) confirmed that the dyadic data followed an indistinguishable dyadic pattern. Bootstrap mediation tests with 95% confidence intervals (5,000 resamples) revealed significant indirect effects of family function (FACES-II) on self-care ability (SCHFI) through symptom perception (HFSPS) and caregiving contribution (EHFScBS-C), as all 95% CIs excluded 0 (Table 5).

(1) Actor effects (patient perspective).

**Table 5:** Mediation effects of family function on self-care ability via symptom perception and caregiving contribution.

Effect type	Path	β	SE	95%CI	Effect proportion
Actor effect					
Direct effect	FACES-II-P → SCHFI	0.182	0.043	0.098–0.271	38.5%
Mediation effect 1	FACES-II-P → HFSPS → SCHFI	0.167	0.058	0.048–0.281	35.3%
Mediation effect 2	FACES-II-P → EHFScBS-C → SCHFI	0.124	0.045	0.033–0.215	26.2%
Total effect		0.473	0.042	0.382–0.549	100%
Partner effect					
Direct effect	FACES-II-S → SCHFI	0.191	0.047	0.101–0.286	40.0%
Mediation effect 1	FACES-II-S → HFSPS → SCHFI	0.154	0.057	0.041–0.269	32.3%
Mediation effect 2	FACES-II-S → EHFScBS-C → SCHF	0.132	0.049	0.036–0.228	27.7%
Total effect		0.477	0.041	0.391–0.553	100%

Total effect of patient-reported family function (FACES-II-P) on self-care ability: *β* = 0.473 (95% CI: 0.382–0.549). Direct effect: FACES-II-P → SCHFI (*β* = 0.182, proportion: 38.5%). Indirect effects: Path 1: FACES-II-P → HFSPS → SCHFI (*β* = 0.167, proportion: 35.3%). Path 2: FACES-II-P → EHFScBS-C → SCHFI (*β* = 0.124, proportion: 26.2%). The total indirect effect proportion is 61.5%.

(2) Partner effects (spouse perspective).

The total effect of spouse-reported family function (FACES-II-S) on patient self-care ability is: *β* = 0.477 (95% CI: 0.391–0.553). The direct effect is: FACES-II-S → SCHFI (*β* = 0.191, proportion: 40.0%). The indirect effects are as follows: Path 1: FACES-II-S → HFSPS → SCHFI (*β* = 0.154, proportion: 32.3%). Path 2: FACES-II-S → EHFScBS-C → SCHFI (*β* = 0.132, proportion: 27.7%). The total indirect effect proportion is 60.0%.

## 4 Discussion

In this study, the family cohesion and adaptability scores of patients with CHF and their spouses were (102.36 ± 18.24) and (98.75 ± 16.83) respectively, indicating a moderately low level. This may be associated with the persistent stress brought by CHF as a progressive chronic disease (17). The significant decline in daily activity tolerance among patients with NYHA class II–III may lead to a reduction in shared family activities, which could further affect the routine interactions that serve as a vehicle for emotional bonding (18). At the same time, long-term medical expenses may create financial pressure, which could not only strain family resources but also trigger anxiety about the future, thereby potentially compressing the space for emotional expression among family members (19). The total score for symptom perception in CHF patients was 52.71 ± 12.38, reflecting a moderate level of distress, which aligns with the patients’ cardiac functional status (20). The mean left ventricular ejection fraction (LVEF) in this cohort was 42.5 ± 6.7%, suggesting impaired cardiac function. Reduced pumping capacity can exacerbate issues such as systemic hypoxia and inadequate perfusion, directly aggravating core symptoms like dyspnea and fatigue. Patients with NYHA class III accounted for 34.9% of the sample, and their symptom scores were 28.7% higher than those of class II patients. Taken together, these findings suggest that a higher NYHA functional class, indicating greater limitations in physical activity, is associated with more intense symptom perception (21, 22). The total self-care ability score of CHF patients in this study was 155.73 ± 43.30, also at a moderately low level, which may be influenced by comorbidities and healthcare experiences. In this study, 36.8% of patients had comorbid diabetes, requiring concurrent management of two chronic conditions, which likely increases the complexity of self-management (23). Additionally, patients had an average of 2.1 ± 1.2 hospitalizations in the past year. Frequent hospitalizations are typically associated with poorer self-care ability (24, 25), suggesting that repeated medical interventions may impact patients’ confidence and behavioral patterns in autonomous disease management.

Correlation analysis revealed a moderate positive correlation between patient- and spouse-reported family functioning (*r* = 0.462, *p* < 0.01). This finding aligns with the concept of “couple cognitive congruence” within Olson’s Family Systems Theory (26). One possible explanation is that shared family circumstances, such as financial stress and access to healthcare resources, exert a synchronous influence on both parties’ evaluations. In families characterized by high cohesion (27, 28), positive interactions may reinforce favorable perceptions of family functioning. Conversely, in families with poorer adaptability (29), maladaptive conflict resolution patterns might lead both members to form more negative evaluations. Both patient and spouse family functioning scores showed significant negative correlations with the patient’s symptom perception (*r* = −0.521 and *r* = −0.487, respectively), suggesting that family functioning may be an important correlate of symptom burden. Mechanistically, families with high adaptability are more likely to establish structured symptom monitoring routines (e.g., daily weight checks), thereby aiding symptom management (30). Furthermore, families with high cohesion, through emotional support, may help patients reduce catastrophic interpretations of symptoms (31)—for instance, reframing “shortness of breath” as “manageable fatigue” rather than a sign of “disease deterioration.” This is consistent with Leventhal’s Common-Sense Model of symptom perception, which posits that the social environment shapes cognitive appraisal (32). Symptom perception demonstrated a strong negative correlation with self-care ability (*r* = −0.612, *p* < 0.01), representing the strongest bivariate association in this analysis. This reflects the potential “symptom distress-behavioral limitation” cycle: physiological symptoms like dyspnea and fatigue directly limit the physical capacity for self-care activities (33), while the anxiety provoked by these symptoms may further erode the patient’s motivation to learn and adhere to self-care regimens. Spousal care contribution was strongly correlated with both family functioning (*r* = 0.538) and patient self-care ability (*r* = 0.597). This indicates that a spouse’s positive perception of family functioning is associated with greater care contribution, and that specific supportive behaviors from the spouse (e.g., medication reminders) are, in turn, linked to better patient self-care. Taken together, these interconnections sketch a plausible interactive pattern: positive family functioning perceptions may promote spousal care behaviors, which could then be associated with enhanced patient self-care by alleviating symptom burden or providing direct support. Improvements in patient self-care may, conversely, reinforce the spouse’s sense of caregiving efficacy (34).

Our APIMeM analysis revealed differentiated pathways linking family functioning in CHF patient-spouse dyads to patient self-care ability. Specifically, the patient’s own pathway operated more through the mediation of symptom perception, whereas the spouse’s pathway operated predominantly through their care contribution. This provides a more nuanced perspective on the interactive processes in heart failure family management. In the actor effects (individual pathways), the contribution of the symptom perception pathway (35.3%) was higher than that of the care contribution pathway (26.2%). This suggests that, for the patient, their perceived family functioning may be linked to self-care behaviors more through the regulation of their own symptom appraisal (35). For example, emotional support in a highly cohesive family may help the patient reframe “dyspnea” from a “sign of deterioration” to a “manageable response,” potentially reducing symptom interference with self-care behaviors such as medication adherence. This indicates that interventions targeting patients might prioritize adjusting symptom cognition. In the partner effects (cross-person pathways), the spouse’s family functioning was linked to the patient’s self-care ability mainly through the spouse’s own care contribution (27.7% contribution), slightly outweighing the path through influencing patient symptom perception. This may stem from the spouse’s typical role as a “medical behavior collaborator” in the family (6). A spouse with higher family functioning is more likely to translate family responsibility into concrete supportive actions, such as checking medication records, and this structural support may directly influence patient self-care. Notably, the association between spouse family functioning and the alleviation of patient symptom perception (*β* = −0.49) was slightly weaker than that of the patient’s own family functioning (*β* = −0.52). A cautious interpretation is that a spouse’s excessive focus on symptom inquiry may inadvertently reinforce the patient’s illness identity (36). Therefore, support for spouses should perhaps emphasize effective caregiving skills over solely providing emotional comfort. Furthermore, the direct effects in the model remained significant (38.5% for the patient pathway, 40.0% for the spouse pathway), indicating that family functioning may also be independently associated with self-care ability beyond the aforementioned mediating pathways. This implies that the sense of security provided by a stable, well-functioning family environment itself may serve as a psychological resource for sustaining self-care behavior. This extends the application of family systems theory in heart failure management, suggesting that family functioning is not only a starting point but may also itself be an important protective factor.

In summary, the pattern of associations observed in this study suggests that when designing family-based heart failure support programs in the future, a differentiated strategy could be considered: for patients, the focus could be on managing and reframing symptom cognition; for spouses, the emphasis could be on enhancing their supportive capabilities in specific medical behaviors. Through such a coordinated pathway, intervention effectiveness might be further improved. Of course, these intervention ideas, derived from cross-sectional association analysis, remain preliminary and hypothetical; their effectiveness must be tested in future longitudinal or interventional studies. This study has several limitations. First, the cross-sectional design precludes causal inference, and the directionality of the pathways discussed awaits confirmation from prospective research. Second, all data were self-reported; although tests for common method bias were conducted, potential bias cannot be ruled out. Future studies could incorporate objective indicators, such as medication adherence records or rehospitalization rates, for validation. Third, the sample was drawn from a single center and consisted predominantly of patients with NYHA class II–III heart failure, which necessitates caution when generalizing the findings. Finally, although the model controlled for several key variables, other potential confounders—such as detailed comorbidity profiles and socioeconomic status—may not have been fully accounted for.

## 5 Conclusion

Based on the Actor-Partner Interdependence Mediation Model (APIMeM), this study explored the associations among family functioning, symptom perception, caregiving contribution, and patient self-care ability in dyads of patients with chronic heart failure and their spouses. The results suggest that family functioning may be differentially linked to patient self-care through dual pathways: the patient’s own pathway operates more through the mediation of symptom perception, while the spouse’s pathway is transmitted mainly via their caregiving contribution. This reflects a potentially complementary functional pattern between spouses in disease management. Building on prior dyadic care research in heart failure, this study integrates family systems and symptom perception theories to elucidate the nuanced mechanisms linking family functioning to self-care behavior via both intrapersonal and cross-person pathways. It provides further empirical evidence for understanding spousal collaborative management within a specific cultural context. Accordingly, clinical practice could consider treating the patient-spouse dyad as a collaborative unit and implementing tiered support: for patients, focus on adjusting symptom cognition; for spouses, strengthen specific caregiving skills; and for the couple, emphasize improving family adaptability and communication quality. Future research should employ longitudinal or intervention designs to further examine the temporal sequence and generalizability of these associations.

## Data Availability

The original contributions presented in the study are included in the article/supplementary material, further inquiries can be directed to the corresponding author.
